# Suicidal Risk and Adverse Social Outcomes in Adulthood Associated with Child and Adolescent Mental Disorders

**DOI:** 10.1177/07067437211055417

**Published:** 2021-11-19

**Authors:** Mariette J. Chartier, James M. Bolton, Okechukwu Ekuma, Natalie Mota, Jennifer M. Hensel, Yao Nie, Chelsey McDougall

**Affiliations:** 1 50023 Department of Community Health Sciences, Rady Faculty of Health Sciences, 8664 University of Manitoba, Winnipeg, Canada; 2 Department of Psychiatry and Community Health Sciences, Rady Faculty of Health Sciences, 8664 University of Manitoba, Winnipeg, Canada; 3 Department of Community Health Sciences, Rady Faculty of Health Sciences, 8664 University of Manitoba, Winnipeg, Canada; 4 Department of Clinical Health Psychology, 8664 University of Manitoba, Winnipeg, Canada; 5 Department of Psychiatry, Rady Faculty of Health Sciences, 8664 University of Manitoba, Winnipeg, Canada; 6 Department of Community Health Sciences, Rady Faculty of Health Sciences, 8664 University of Manitoba, Winnipeg, Canada; 7 Department of Community Health Sciences, Rady Faculty of Health Sciences, 8664 University of Manitoba, Winnipeg, Canada

**Keywords:** Anxiety disorders, self-medication, suicide, suicidal behavior, epidemiology, anxiety disorders

## Abstract

**Objective:**

The life course of children and adolescents with mental disorders is an important area of investigation, yet it remains understudied. This study provides a first-ever comprehensive examination of the relationship between child and adolescent mental disorders and subsequent suicidal and adverse social outcomes in early adulthood using population-based data.

**Methods:**

De-identified administrative databases were used to create a birth cohort of 60,838 residents of Manitoba born between April 1980 to March 1985 who were followed until March 2015. Unadjusted and adjusted hazard ratios (aHRs) and odds ratios (aORs) were calculated to determine associations between physician-diagnosed mental disorders in childhood or adolescence and a range of adverse early adulthood (ages 18 to 35) outcomes.

**Results:**

Diagnoses of mood/anxiety disorders, attention-deficit hyperactivity disorder, substance use disorder, conduct disorder, psychotic disorder, personality disorders in childhood or adolescence were associated with having the same diagnoses in adulthood. These mental disorder diagnoses in childhood/adolescence were strongly associated with an increased risk of suicidal behaviors and adverse adult social outcomes in adulthood. Similarly, suicide attempts in adolescence conferred an increased risk in adulthood of suicide death (aHR: 3.6; 95% confidence interval [CI]: 1.9-6.9), suicide attempts (aHR: 6.2; CI: 5.0–7.6), social housing use (aHR: 1.7; CI 1.4–2.1), income assistance (aHR: 1.8; CI 1.6–2.1), criminal accusation (aHR: 2.2; CI 2.0–2.5), criminal victimization (aHR:2.5; CI 2.2–2.7), and not completing high school (aOR: 3.1; CI: 2.5–3.9).

**Conclusion:**

Mental disorders diagnosed in childhood and adolescence are important risk factors not only for mental disorders in adulthood but also for a range of early adult adversity. These findings provide an evidence-based prognosis of children's long-term well-being and a rationale for ensuring timely access to mental health services. Better population-level mental health promotion and early intervention for children and adolescents with mental disorders are promising for improving future adult outcomes.

## Abrégé

**Objectif:**

Le parcours de vie des enfants et des adolescents souffrant de troubles mentaux est un domaine important d’investigation, et pourtant, il demeure sous-étudié. La présente étude offre pour la première fois un examen exhaustif de la relation entre les troubles mentaux de l’enfant et de l’adolescent et subséquemment, des résultats suicidaires et sociaux indésirables au début de l’âge adulte à l’aide de données dans la population.

**Méthodes:**

Des bases de données administratives dépersonnalisées ont été utilisées pour créer une cohorte de naissance de 60 838 résidents du Manitoba nés entre avril 1980 et mars 1985 qui ont été suivis jusqu’en mars 2015. Des rapports de risques non ajustés et ajustés (RRa) et des rapports de cotes (RCa) ont été calculés pour déterminer les associations entre les troubles mentaux diagnostiqués par un médecin dans l’enfance ou l’adolescence et une série de résultats indésirables du début de l’âge adulte (de 18 à 35 ans).

**Résultats:**

Les diagnostics de troubles de l’humeur/anxieux, du trouble de déficit de l’attention avec hyperactivité, du trouble d’utilisation de substances, du trouble des conduites, du trouble psychotique, des troubles de la personnalité dans l’enfance ou l’adolescence étaient associés aux mêmes diagnostics à l’âge adulte. Ces diagnostics de troubles mentaux dans l’enfance/l’adolescence étaient fortement associés à un risque accru de comportements suicidaires et de résultats sociaux indésirables à l’âge adulte. De même, les tentatives de suicide à l’adolescence conféraient un risque accru de décès par suicide à l’âge adulte (RRa 3,6; Intervalle de confiance (IC) à 95% 1,9 à 6,9), de tentatives de suicide (RRa 6,2; IC à 95% 5,0 à 7,6), de recours au logement social (RRa 1,7; IC à 95% 1,4 à 2,1), d’assistance au revenu (RRa 1,8; IC à 95% 1,6 à 2,1), d’accusation criminelle (RRa 2,2; IC à 95% 2,0 à 2,5), de victimisation criminelle (RRa 2,5; IC à 95% 2,2 à 2,7) et de cours secondaire non terminé (RCa 3,1; IC à 95% 2,5 à 3,9).

**Conclusion:**

Les troubles mentaux diagnostiqués dans l’enfance et l’adolescence sont des facteurs de risque importants non seulement pour les troubles mentaux à l’âge adulte mais aussi pour une série d’épreuves en début d’âge adulte. Ces résultats offrent un pronostic fondé sur des données probantes du bien-être à long terme des enfants, et justifient d’assurer un accès en temps opportun aux services de santé mentale. Une meilleure promotion de la santé mentale dans la population et une intervention précoce pour les enfants et les adolescents souffrant de troubles mentaux sont prometteuses d’une amélioration future des résultats des adultes.

## Introduction

Mental disorders in children and adolescents are highly prevalent^1^ and are associated with emotional distress and considerable interference with academic success, relationships, and eventually participation in the workforce.^2^ A US epidemiological study reported that 13.0% of boys and 9.4% of girls experienced a mental disorder with severe impairment and half of the children identified received no specialty mental health care.^3^ Furthermore, the age of onset of most mental disorders is in childhood, with symptoms often persisting into adulthood.^4^ A growing body of research now suggests that childhood and adolescent mental disorders are associated with adverse outcomes in adulthood.^5–8^ A US report on youth mental health stresses the importance of keeping children and youth mentally healthy and on mental illness prevention, instead of waiting until an illness is well established and has caused considerable suffering.^9^

Our understanding of the link between childhood and adolescent mental disorders and adverse adult outcomes is limited, particularly in the Canadian context. A Canadian study based on the National Population Health Survey reported associations between depression in adolescence and later depression, poor self-rated health and low social support in adulthood.^10^ A recent meta-analysis suggested that depression in adolescence was also associated with unemployment, failure to complete high school and parenthood.^11^ The majority of existent studies have relied on clinical samples and surveys that are prone to a number of biases including selection, reporting, and recall biases. Reaching broad populations with surveys is challenging and vulnerable participants are particularly prone to be lost to follow-up.^12^ When surveyed about past health concerns, participants may bias the study by not recalling their health histories or not reporting because of social desirability.^13,14^ Many studies have also relied on survey instruments that identify emotional and behavioral symptoms but may not have met the diagnostic criteria for a mental disorder.

A recent study using the Danish Psychiatric Registry addressed some of these biases and found that individuals with a history of childhood and adolescent mental disorders were five times more likely to be referred for psychiatric treatment in adulthood.^15^ However, this study did not control for confounding factors such as socio-economic status or child adversity factors that could explain the association. To our knowledge, no previous studies have examined a broad range of mental disorders, suicidal behaviors, and social outcomes for a cohort from birth into adulthood using administrative data which address the sampling and data collection challenges described and accounts for demographic and social confounders. Understanding the life course of children and adolescents diagnosed with mental disorders is an important area for investigation, since it could directly inform policy and practice that could prevent these later adverse adult outcomes.^9^

The objective of the current study was to use population-based administrative databases to follow a birth cohort of individuals with and without childhood and adolescent mental disorders to examine the long-term associations with suicidal risk and adverse adult social outcomes. The extensive collection of health, justice, education, and social services databases available in Manitoba provide the ability to examine a range of important childhood factors and life events not previously studied. Given prior research, we hypothesized that individuals with a history of childhood or adolescent mental disorders would have a higher risk of suicidal behaviors, social services use, criminal accusations and victimizations, and failure to complete high school in early adulthood compared to those without such a history.

## Methods

### Study Overview

We built a birth cohort of Manitoba residents born between April 1980 to March 1985 and followed them to the end of study period where data were available, March 2015. The cohort was constructed from de-identified administrative databases from the Manitoba Population Research Data Repository housed at the Manitoba Centre for Health Policy (MCHP). Given the birth cohort used data collected over a five-year period, the youngest cases were 30 and the oldest, 34 years old by the end of the follow-up period. This study was approved by the University of Manitoba research ethics board and the Health Information Privacy Committee of Manitoba Health, Seniors and Active Living. Given that the administrative data are de-identified, we have not obtained individual informed consents.

### Study Population

**Figure 1.**
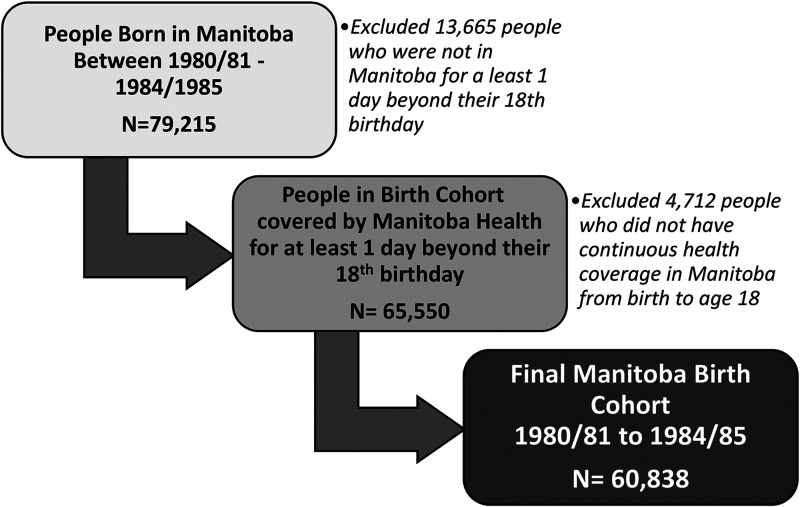
Flow chart of birth cohort (April 1, 1980/81–1984/85) formation.

The birth cohort consisted of 60,838 residents of Manitoba, a province in Central Canada with a population of 1.3 million people. Manitoba has a publicly financed health care system and maintains databases on all its citizens dating back to the 1970s. The vast majority of adolescents attend publicly funded schools. Of the 79,215 people born in Manitoba during the cohort inclusion period, 13,665 were excluded because they were not covered by Manitoba Health for at least one day beyond their 18th birthday and another 4,712 were excluded due to lack of continuous health coverage from birth to age 18 (Figure 1). Our final birth cohort included 60,838 people who had lived continuously in Manitoba from birth to age 18 and had lived in Manitoba for at least one day after their 18th birthday.

### Data Sources

The Data Repository is one of the most extensive linkable person-level database holdings in world, with over 90 databases including health, social, education, and justice data.^16–18^ These data are collected on virtually all Manitoba residents (over 99%) and are linkable through a scrambled health information number, providing a de-identified longitudinal health and social profile for the population. Datasets from different sources were used to create the study variables: physician billing claims, hospital records, and prescription database (child and adult mental disorders and suicide attempts); Manitoba Health Insurance Registry (age, sex, urbancity, family size, two parent family, and cohort construction); Canada Census (area-level income); Child and Family Services (child welfare); Vital Statistics (suicide deaths); Tenant Management System (social housing); Employment and Income Assistance (income assistance); Prosecutions Management Information System (criminal accusations and victimizations); and Education databases (high-school graduation).

### Diagnosed Childhood and Adolescent Mental Disorders

We defined childhood/adolescent mental disorders through physician billings claims, hospital records, and prescription data. These disorders were based on ICD-9 CM and ICD-10 CA diagnostic codes (See Table 1) and coded using established definitions. These diagnostic definitions have been used extensively in other studies.^19–21^ The list of diagnosed mental disorders include the following: mood or anxiety disorders, attention-deficit hyperactivity disorder (ADHD), substance use disorders, conduct disorder, psychotic disorders, personality disorders, and any mental disorder (at least one of the previous diagnoses). We also extracted hospital records of suicide attempts. Personality disorders were included to be consistent with our definitions of adult mental disorders and due to emerging evidence of their prevalence in adolescence.^22^ Mental disorder diagnoses found for children under four years of age were excluded due to the challenges of reliable diagnosis in pre-school children and to be consistent with previous Canadian epidemiologic studies.^2^

**Table 1.** Definitions of Childhood/Adolescent Mental Disorders, Early Adult Outcomes and Covariates.

Early adult outcomes	Definition
Mood and anxiety disorders	One or more hospitalizations with a diagnosis for depressive disorder, affective psychoses, neurotic depression, adjustment reaction, bipolar disorder, an anxiety state, phobic disorders or obsessive-compulsive disorders: ICD-9-CM codes 296, 311, 309, 300 or ICD-10-CA codes F30, F31, F32, F33, F34, F38, F40, F41.0, F41.1, F41.2, F41.3, F41.8, F41.9, F42, F43, F53.0; OR Two or more physician visits with a diagnosis for depressive disorder or affective psychoses, adjustment reaction or for anxiety disorders (including dissociative and somatoform disorders): ICD-9-CM codes 296, 311, 309, 300
Attention deficit hyperactivity disorder (ADHD)	One or more hospitalizations with a diagnosis of hyperkinetic syndrome in one fiscal year: ICD-9-CM code 314 or ICD-10-CA code F90; OR One or more physician claims with a diagnosis of hyperkinetic syndrome in one fiscal year: ICD-9-CM code 314; OR Two or more prescriptions for ADHD drugs without a diagnosis in the same fiscal year of: – conduct disorder: ICD-9-CM code 312 or ICD-10-CA codes F63, F91, F92; OR – disturbance of emotions: ICD-9-CM code 313 or ICD-10-CA codes F93, F94; OR – cataplexy/narcolepsy: ICD-9-CM code 347 or ICD-10-CA code G47.4; OR One prescription for ADHD drugs in one fiscal year with a diagnosis of hyperkinetic syndrome in the previous three years: ICD-9-CM code 314 or ICD-10-CA code F90. The lists of ADH medication used was based on Brownell et al. (2012) found here: http://appserv.cpe.umanitoba.ca/concept/MB_Kids_2012_ADHD_DIN_List_DPIN.pdf
Substance use disorders	One or more hospitalizations with a diagnosis for alcohol or drug psychoses, alcohol or drug dependence, or nondependent abuse of drugs: ICD-9-CM codes 291, 292, 303, 304, 305 or ICD-10-CA codes F10-F19, F55, Z50.2, Z50.3; OR One or more physician visits with a diagnosis for alcohol or drug psychoses, alcohol or drug dependence, or nondependent abuse of drugs: ICD-9-CM codes 291, 292, 303, 304, 305.
Conduct disorder	One or more hospitalizations with a diagnosis of conduct disorder: ICD-9-CM code 312 or ICD-10-CA codes F91 (all except F91.3 (oppositional disorder)); OR One or more physician visits with a diagnosis of conduct disorder: ICD-9-CM code 312.
Psychotic disorders	One or more hospitalizations with a diagnosis of psychotic disorders: ICD-9-CM codes 295, 297, 298 or ICD-10-CA codes F11.5, F12.5, F13.5, F14.5, F15.5, F16.5, F18.5, F19.5, F20, F22, F23, F24, F25, F28, F29; OR One or more physician visits with a diagnosis of psychotic disorders: ICD-9-CM codes 295, 297, 298.
Personality disorders	One or more hospitalizations with a diagnosis for personality disorders: ICD-9-CM code 301 or ICD-10-CA codes: F21, F60, F61, F62, F69; OR One or more physician visits with a diagnosis of personality disorders: ICD-9-CM code 301.
Early adult outcomes (con’t)	Definition
Hospitalizations for attempted suicide	One or more hospitalizations with a diagnosis for self-inflicted injury or poisoning: ICD-9-CM codes E950-E959 or ICD–10–CA codes X60–X84; OR One or more hospitalizations with a diagnosis code for poisoning of undetermined intent, injury of undetermined intent, or accidental poisoning, only if there is a mental illness code during the hospital stay: ICD-9-CM codes E850-E854, E858, E862, E868 or ICD–10–CA codes Y10–Y34, T39, T40, T42.3, T42.4, T42.7, T43, T50.9, T58, X44, X46, X47.
Suicide	Suicide among adults was defined as having a death record in Vital Statistics data with the following listed as the primary cause of death: Accidental poisoning: ICD-9-CM codes E8509, E8529, E8502, E8629, E8689, or ICD-10-CA codes X40-X42, X46, X47 Self-inflicted poisoning: ICD-9-CM codes E950-E952, or ICD-10-CA codes X60-X69 Self-inflicted injury: ICD-9-CM codes E953, E954, E955, E956, E957, E958, or ICD-10-CA codes X70-X84 Late effects of self-inflicted injury: ICD-9-CM code E959, or ICD-10-CA codes Y10-Y12, Y16, Y17, Y87.0
Not graduating from high school	Individuals who have not completed grade 12 as determined by the Department of Education data.
Criminal accusation	Individuals who have had contact with the justice system and are identified as having been accused of a crime using the PRISM (Prosecutions Information and Scheduling Management) database.
Criminal victimization	Individuals who have had contact with the justice system and are identified as having been a victim of a crime using the PRISM (Prosecutions Information and Scheduling Management) database.
Receiving income assistance	Individuals who receive financial assistance, administered through Manitoba's Employment and Income Assistance program, to meet basic personal and family needs.
Living in social housing	People living in social housing that is owned and directly managed by Manitoba Housing.
Childhood/adolescent covariates	Definition
Area-level income quintile	An income quintile is a measure of neighborhood socioeconomic status that divides the population into five income groups (from lowest income to highest income) so that approximately 20% of the population is in each group. Measured using 2001 Census data, data that was closest to the cohort member's teenage years.
Urban or rural residence	Individuals living in Winnipeg or Brandon have an urban residence. Individuals living elsewhere in Manitoba have a rural residence. Measured using postal code when cohort member was 17 years old.
Two parent family	This family covariate was coded as present if cohort member was from a two parent family at some point between birth and age 12 years. Two parent family is defined as such when two individuals have registered their marital union with Manitoba Health and have one or more children under the age of 18.
Number of children in family	The number of children registered under the “family head” in the Manitoba Health Registry. This family covariate was measured when cohort member was 17 years old.
Any diagnosis of maternal mental illness	Children whose mother had at least one diagnosis of mood and anxiety disorders, substance use disorders, psychotic disorders (including schizophrenia), or personality disorders. This family covariate was coded as present if mother had a diagnosis at some point between the cohort member's birth and age 17 years.
In care by child welfare	Children who have been removed from the care of their original families because of a situation where authorities have deemed their family unable or unfit to look after them properly. In some cases, children are voluntarily placed into care by their parents or guardians. Children can come into care for a variety of reasons including abuse, neglect, illness, death of a parent, addiction issues or conflicts in their family, disability, or emotional problems. This covariate was coded as present if the cohort member was removed from home between birth and age 17 years.

### Demographic and Social Childhood Covariates

We included the following demographic and social covariates to control for their possible confounding effects: sex, area-level income, urban (vs. rural), two parent family, number of children in the family, maternal mental disorders, and in care of child welfare during childhood. A complete description of these covariates is included in Table 1.

### Early Adult Outcomes

Early adult outcomes (from 18 to 35 years), examined and defined in Table 1, included the same mental disorders examined in childhood/adolescence as well as suicide and attempted suicide. The following social outcomes were also included: failure to complete high school, accused of a crime, victim of a crime, receiving income assistance, and living in social housing. Each outcome was categorized as being present or not during the follow-up period.

### Analytic Strategy

In order to take a preliminary look at the childhood/adolescent and adult outcome variables, we calculated the number and percentage of each childhood covariate and each adult outcome for those with a diagnosed mental disorder in childhood or adolescence and for those without. We conducted Chi-square and t-tests to test for differences between the two groups.

Next, unadjusted and adjusted hazard ratios and odds ratios with 95% confidence intervals were calculated to determine the associations between mental disorders in childhood or adolescence and adverse early adult outcomes. Specifically, we used Cox proportional hazard regression to test a long-term association between childhood/adolescent mental disorders and adverse outcomes over the course of early adult years. This method allowed for follow-up of the entire cohort into early adulthood and adjusted for those who were no longer in the cohort because of death or having moved out of the province. Hence, we modeled time to first record of each of the early adult outcomes (see Table 1). Schoenfeld's residuals and covariates interaction with log of time were used to test for violation of proportional hazard assumptions. Given that high-school graduation generally occurs in the late teen years and not evenly over the course of early adulthood, it was not appropriate to use Cox proportional hazard regression. Logistic regression was therefore used to determine if people with childhood/adolescent mental disorders were less likely to graduate from high school compared to those without mental disorders. Each outcome was modeled with and without adjustments for demographic and social covariates as described earlier. Analyses were done using SAS® version 9.4.^23^

## Results

### Birth Cohort Description

Of the 60,388 people in the cohort, 16.5% (*n* = 10,040) were diagnosed with at least one of the mental disorders at some point during their childhood or adolescence. The mean age of onset in years for these disorders diagnosed in childhood or adolescence was as follows: mood/anxiety disorders, 14.2; ADHD, 11.7; substance use disorders, 15.6; conduct disorder, 11.5; psychotic disorders, 14.1; personality disorders, 14.5. Table 2 shows differences in the childhood demographic and social covariates between those with and without mental disorders. Compared to individuals with no diagnosed childhood/adolescent mental disorders, those who were diagnosed were more likely to be from low-income areas (58.5% vs. 54.1%), live in urban areas (60.2% vs. 50.0%), have a mother with a history of mental illness (74.3% vs. 57.4%), and have been in care of child welfare (12.8% vs. 2.3%). They were less likely to be male (50.0% vs. 51.5%), from a two-parent family (55.2% vs. 70.2%), and from a large family (22.2% vs. 24.0%). In early adulthood, the group with a history of childhood/adolescent mental disorders also had a higher proportion of suicide attempts (3.4% vs. 0.85%), suicide deaths (0.54% vs. 0.18%), criminal accusations (26.0% vs. 14.1%), criminal victimizations (38.8% vs. 24.1%), received income assistance (17.3% vs. 6.6%), lived in social housing (5.8% vs. 2.5%), and not completed high school (58.1% vs. 49.5%) compared to those not diagnosed.

**Table 2.** Number and Percentage of Individuals With and Without Any Childhood/Adolescent Mental Disorders by Childhood/Adolescent Factors and by Adverse Early Adult Outcomes.

	Any mental disorder^1^ (*n* = 10,040)		No mental disorders (*n* = 50,798)		*p*-value
	Number	Percent	Number	Percent	
Childhood/adolescent factors					
Males	5,017	49.97	26,178	51.53	0.0042
Lowest income quintiles^2^	5,877	58.54	27,482	54.10	<.0001
Urban	6,044	60.20	25,374	49.95	<.0001
Two parent family	5,546	55.24	35,659	70.20	<.0001
Maternal mental health diagnosis	7,459	74.29	29,174	57.43	<.0001
4 or more children in family	2,230	22.21	12,170	23.96	0.0002
Being in care of child welfare	1,284	12.79	1,178	2.32	<.0001
Adverse early adult outcomes					
Hospitalizations for attempted suicide	344	3.43	434	0.85	<.0001
Deaths by suicide	54	0.54	91	0.18	<.0001
Victim of a crime	2,610	26.00	7,183	14.14	<.0001
Accused of a crime	3,897	38.81	12,218	24.05	<.0001
Income assistance	1,737	17.30	3,347	6.59	<.0001
Social housing	584	5.82	1,250	2.46	<.0001
Failure to complete high school	5,830	58.07	25,147	49.50	<.0001

^1^ Any mental disorder includes the following disorders: mood and anxiety disorders, ADHD, substance use disorders, conduct disorders, psychotic disorders (including schizophrenia), and personality disorders.

^2^ Includes the lowest two income quintiles in rural and urban regions.

### Adult Mental Disorders

Table 3 shows that a higher proportion of individuals diagnosed with a childhood/adolescent mental disorder received that same diagnosis in early adulthood compared to those not diagnosed in childhood/adolescence. For example, 69.8% (3,635) of those diagnosed with mood and anxiety disorders in childhood/adolescence also had a mood and anxiety disorder diagnosis over the course of their early adulthood compared to 34.2% (19,010) of those with no diagnosis in childhood/adolescence. The unadjusted and adjusted hazard ratios show the strength of the association and suggest that childhood /adolescent mental disorders persist into adulthood. For example, those diagnosed with a substance use disorder in childhood/adolescence were over three times more likely to also be diagnosed as a young adult (adjusted hazard ratio [aHR]: 3.35, 95% confidence interval [CI]: 3.12–3.59) compared to those not diagnosed with a substance use disorder in childhood/adolescence.

**Table 3.** Associations Between Specific Childhood/Adolescent Mental Disorders and the Same Mental Disorder in Adulthood.

Specific mental disorder	Among those with history of a specific childhood/ adolescent disorder, % (*n*) of adults with the same disorder	Among those with NO history of a specific childhood/ adolescent disorder, % (*n*) of adults with the disorder	Unadjusted hazard ratio (95% CI)	Adjusted^1^ hazard ratio (95% CI)
Mood and anxiety disorders	69.8 (3,635)	34.2 (19,010)	**3.11** **(** **3.01–3.23)**	**2.52** **(** **2.43–2.62)**
Attention-deficit hyperactivity disorder (ADHD)	9.2 (211)	1.4 (807)	**7.35** **(** **6.31–8.55)**	**5.43** **(** **4.62–6.39)**
Substance use disorders	50.0 (978)	13.0 (7,634)	**5.20** **(** **4.86–5.56)**	**3.35** **(** **3.12–3.59)**
Conduct disorder	2.6 (79)	0.3 (199)	**7.49** **(** **5.77–9.72)**	**5.70** **(** **4.29–7.57)**
Psychotic disorders (including Schizophrenia)	39.5 (145)	1.7 (1,051)	**31.79** **(** **26.72–37.82)**	**20.84** **(** **17.34–25.04)**
Personality disorders	23.9 (121)	2.3 (1,398)	**11.84** **(** **9.83–14.25)**	**6.39** **(** **5.24–7.79)**
Any mental disorder^2^	65.8 (6,602)	39.2 (19,934)	**2.34** **(** **2.27–2.40)**	**2.13** **(** **2.07–2.19)**

**Bold values** indicate a statistically significant association (*p* < 0.05).

^1^ Adjusted for sex, income quintiles, urbanicity, two parent family, number of children in the family, maternal mental health diagnosis, and being taken into care during childhood.

^2^ Any mental disorder includes the following disorders: mood and anxiety disorders, ADHD, substance use disorders, conduct disorders, psychotic disorders (including schizophrenia), and personality disorders.

### Adult Suicidal Risk and Adverse Social Outcomes

The estimates in Table 4 suggest moderate and strong associations between childhood/adolescent mental disorders and adult suicidal risk and adverse social outcomes. Adjusting for other childhood factors attenuated these associations; the vast majority remained statistically significant.

**Table 4.** Adjusted^1^ and Unadjusted Associations Between Specific Childhood Mental Disorders and Early Adult Adverse Outcomes.

		Hazard ratio (95% confidence intervals)						Odds ratio (95% CI)
Childhood mental disorders	Model	Suicide	Suicide attempts	Income assistance	Social housing	Criminal accusations	Criminal victimization	Not completing high school
Mood or anxiety disorders	Unadjusted	**2.67** (**1.78–4.01)**	**4.17** (**3.56–4.87)**	**2.83** (**2.64–3.03)**	**2.50** (**2.22–2.81)**	**1.34** (**1.28–1.41)**	**1.89** (**1.79–2.01)**	**1.85** (**1.71–1.99)**
	Adjusted	**2.48** (**1.62–3.82)**	**3.53** (**2.99–4.16)**	**2.15** (**2.00–2.31)**	**1.52** (**1.53–1.72)**	**1.32** (**1.25–1.39)**	**1.44** (**1.36–1.53)**	**1.72** (**1.58–1.88)**
Attention-deficit hyperactivity disorder (ADHD)	Unadjusted	S	**1.81** (**1.36–2.40)**	**2.35** (**2.13–2.61)**	0.90 (0.71–1.14)	**2.15** (**2.02–2.29)**	**1.47** (**1.35–1.61)**	**2.66** (**2.40–2.94)**
	Adjusted	S	**1.88** (**1.40–2.53)**	**2.34** (**2.10–2.60)**	1.20 (0.95–1.51)	**1.45** (**1.36–1.55)**	**1.31** (**1.19–1.43)**	**2.15** (**1.92–2.41)**
Substance use disorder	Unadjusted	**6.61** (**4.32–10.10)**	**9.00** (**7.60–10.66)**	**3.39** (**3.08–3.72)**	**3.77** (**3.25–4.37)**	**3.14** (**2.95–3.33)**	**3.46** (**3.22–3.72)**	**5.52** (**4.90–6.22)**
	Adjusted	**3.58** (**2.26–5.68)**	**4.77** (**3.97–5.73)**	**1.91** (**1.72–2.11)**	**1.57** (**1.35–1.84)**	**2.23** (**2.09–2.38)**	**2.04** (**1.89–2.20)**	**3.45** (**3.01–3.95)**
Conduct disorder	Unadjusted	**2.64** (**1.61–4.32)**	**2.42** (**1.94–3.02)**	**2.31** (**2.11–2.53)**	**1.94** (**1.65–2.27)**	**1.93** (**1.82–2.04)**	**1.69** (**1.57–1.82)**	**2.44** (**2.23–2.68)**
	Adjusted	**1.78** (**1.04–2.97)**	**2.26** (**1.79–2.85)**	**1.94** (**1.76–2.13)**	**1.49** (**1.26–1.75)**	**1.34** (**1.27–1.42)**	**1.33** (**1.23–1.44)**	**1.81** (**1.63–2.01)**
Psychotic disorders (including schizophrenia)	Unadjusted	S	**9.37** (**6.84–12.83)**	**3.99** (**3.28–4.84)**	**2.43** (**1.66–3.56)**	**1.79** (**1.53–2.10)**	**1.61** (**1.31–1.98)**	**3.42** (**2.64–4.44)**
	Adjusted	S	**5.95** (**4.31–8.22)**	**2.48** (**2.04–3.02)**	1.27 (0.87–1.86)	1.13 (0.97–1.33)	1.01 (0.82–1.25)	**2.17** (**1.60–2.93)**
Personality disorders	Unadjusted	S	**7.12** (**5.23–9.68)**	**3.92** (**3.32–4.63)**	**3.13** (**2.34–4.18)**	**2.30** (**2.03–2.60)**	**2.86** (**2.48–3.30)**	**4.02** (**3.23–5.00)**
	Adjusted	S	**4.41** (**3.20–6.07)**	**2.17** (**1.83–2.58)**	**1.40** (**1.04–1.88)**	**1.68** (**1.48–1.91)**	**1.69** (**1.47–1.96)**	**2.49** (**1.94–3.21)**
Any mental disorder^2^	Unadjusted	**3.01** (**2.15–4.22)**	**4.06** (**3.53–4.68)**	**2.81** (**2.65–2.98)**	**2.41** (**2.18–2.66)**	**1.83** (**1.77–1.90)**	**2.00** (**1.91–2.09)**	**2.50** (**2.36–2.65)**
	Adjusted	**2.38** (**1.65–3.41)**	**3.49** (**3.01–4.05)**	**2.28** (**2.14–2.43)**	**1.60** (**1.44–1.78)**	**1.55** (**1.50–1.61)**	**1.58** (**1.50–1.65)**	**2.10** (**1.97–2.24)**
Attempted suicide	Unadjusted	**6.28** (**3.40–11.61)**	**13.92** (**11.38–17.01)**	**3.64** (**3.17–4.19)**	**4.83** (**3.96–5.88)**	**2.78** (**2.53–3.06)**	**4.55** (**4.12–5.02)**	**5.23** (**4.29–6.37)**
	Adjusted	**3.60** (**1.89–6.87)**	**6.15** (**4.96–7.63)**	**1.79** (**1.55–2.07)**	**1.67** (**1.36–2.05)**	**2.23**(**2.02–2.46)**	**2.45** (**2.21–2.71)**	**3.12** (**2.49–3.91)**

**Bold values** indicate a statistically significant association (*p* < 0.05).

^1^ Adjusted for sex, income, urbanicity, two parent family, number of children in family, maternal mental health, and being taken into care during childhood.

^2^ Any mental disorder includes the following disorders: mood and anxiety disorders, ADHD, substance use disorders, conduct disorders, psychotic disorders (including schizophrenia), and personality disorders.

*S* indicates suppressed because of small sample size.

#### Suicidal Risk

Having a childhood/adolescent mental disorder increased the likelihood of both suicide and attempted suicide in adulthood. In adjusted analyses, a childhood/adolescent suicide attempt was strongly associated with a suicide attempt in adulthood (aHR: 6.15, CI: 4.96–7.63), as were adolescent psychotic disorders (aHR: 5.95, CI: 4.31–8.22) and substance use disorders (aHR: 4.77, CI: 3.97–5.73). For suicidal deaths, those with a childhood/adolescent substance use disorder or who were hospitalized for attempted suicide in adolescence were, respectively, 3.58 and 3.60 times more likely to die by suicide in adulthood compared to those with no such history in their childhood or adolescence.

#### Social Services Use

After adjustments for confounding childhood factors, individuals with childhood/adolescent mental disorders were more likely to receive income assistance in adulthood with adjusted hazard ratios ranging from 1.79 to 2.48, compared to those not diagnosed with these disorders in childhood/adolescence. In examining social housing, almost all childhood/adolescent mental disorders were associated with using this service in early adulthood. Compared to individuals with no adolescent history of attempted suicide, those who attempted suicide in adolescence were more likely to live in social housing in early adulthood (aHR: 1.67, CI: 1.36–2.05). However, after adjustments, the association between both ADHD and psychotic disorders and living in social housing were no longer statistically significant, suggesting that other childhood factors explained the association between history of these childhood/adolescent mental disorders and social housing. We note that the hazard ratios are relatively similar across the mental health indicators suggesting that these indicators posed similar risk for increased social services use.

#### Justice System Involvement

Our findings suggest that having a childhood/adolescent mental disorder increased the likelihood of justice system involvement in adulthood. Those with substance use disorders were close to twice as likely to be accused of a crime or be victimized compared to those with no history of childhood/adolescent substance use disorders. The strength of these associations was similar across childhood/adolescent mental disorders for both accusations and victimizations. Unexpectedly, the association between being hospitalized for attempted suicide in adolescence and being criminally accused in early adulthood (aHR: 2.23, CI: 2.02–2.46) was stronger than having a conduct disorder in childhood/adolescence and being criminally accused (aHR: 1.34, CI: 1.27–1.42). It is noteworthy that after adjustments for other childhood factors, no association was found between childhood/adolescent psychotic disorders and justice system involvement.

#### Failure to Complete High School

Having a childhood/adolescent mental disorder was associated with failure to complete high school, even after adjustments for other childhood factors. Individuals with childhood/adolescent substance use disorder or suicidal behaviors were close to three times as likely to not complete high school compared to those without these mental health problems in childhood/adolescence.

## Discussion

The novel contribution of this study is using a population-based cohort to comprehensively examine the long-term association between mental disorders in childhood or adolescence and a range of mental disorders, suicidal behaviors, and social outcomes in early adulthood. Childhood/adolescent mental disorders were associated with an increased risk of adverse early adult outcomes, by two- to four-fold, including mental disorders in adulthood, suicide attempts and deaths, use of income assistance and social housing, criminal accusations and victimizations, and not completing high school. Suicide attempts and substance use disorders were associated with high hazards ratios for adverse outcomes. The relatively smaller hazard ratios observed with mood and anxiety disorders should not be discounted considering their high prevalence among children and adolescents worldwide.^24^ Adjusting for other childhood factors attenuated these associations between mental disorders and adverse adult outcomes, but almost all remained statistically significant.

Our finding that childhood/adolescent mental disorders are associated with higher suicidal risk in adulthood has been previously reported in survey-based studies^7,25^ but not yet in a study using administrative databases. Similar with the current study, a New Zealand longitudinal study reported associations between childhood/adolescent mental health problems and adult mental disorders noting that other childhood factors accounted for part of these relationships.^26–28^ Survey data has shown that half of people reporting mental disorders in adulthood had symptoms before age 14 and three quarters had symptoms before age 24.^29^ Results from a recent Danish study^15^ were in line with the unadjusted estimates in this study, pointing to the importance of accounting for the confounding effects of other childhood factors to understand the unique influence of the childhood/adolescent mental disorders on later adult mental health. Costello and Maughan (2015) summarized the evidence showing an association between childhood depression, ADHD, antisocial behaviors or substance use disorders and adult mental disorders.^8^ Another study reported that childhood emotional and behavioral symptoms were associated with DSM-IV disorders in adulthood, with the exception of attention-deficit hyperactivity problems.^30^

Consistent with this study, others have found long-term social and academic consequences of childhood and adolescent mental disorders. Using data from the Great Smokey Mountain Survey,^7^ the study found associations between childhood/adolescent disorders and increased risk of incarcerations, employment and residence instability, and high school drop-out. Previous research reported that those with childhood mental disorders were less likely to find work and get married^6^ and that conduct disorder in childhood was associated with criminality in adulthood; however, ADHD in childhood was not.^31^ Children with depression performed more poorly academically over time^32^ and young people with childhood mental disorders were between 1.5 to 3.5 times less likely to complete high school.^5^ Finally, Costello and Maughan's review found an association between mental disorders and poor academic outcomes, justice system involvement, and work impairment.^8^

The findings of this study suggest that many mental disorders experienced by the adult population have their roots in childhood, pointing to strengthening all levels of mental health services across the continuum from mental health promotion to treatment. Adolescents hospitalized for suicide attempts appear to be at particularly high risk for adverse adult outcomes, warranting longer-term follow-up. These results are relevant to clinical practice in providing an evidence-based prognosis of children's long-term health and well-being and rationale for screening of mental disorders as well as appropriate and timely access to mental health services. For child and adolescent mood and anxiety disorders, cognitive behavior therapy and interpersonal therapies as well as pharmacological approaches namely selective serotonin reuptake inhibitors have been shown to be effective.^33,34^ The evidence for addressing adolescent substance use disorders is scarcer; however, motivational enhancement therapy and family-based therapies are associated with some effects.^35^ To address barriers to access to child and adolescent mental health services, models such as integrating pediatric behavioural service into primary care should be considered.^36^ The present study also highlights the importance of being attentive to young people's overall academic and social functioning and possible requirements for extra supports of children and adolescents experiencing mental disorders.

Given the high prevalence of mental disorders in Canada and worldwide and the substantial economic and social costs to individuals and to society, a broader approach to population mental health should be considered.^37,38^ School-based universal programs aimed at preventing depression and anxiety disorders are associated with small effect sizes; however, small effect sizes can make big differences at a population level.^39^ Bennett et al. (2015) conducted a systematic review highlighting a number of programs, designed for youth, that have been shown to decrease suicide ideation and attempts.^40^ Colman et al. (2014) provided evidence that depression in adulthood is influenced by an accumulation of factors across the life course starting in early childhood.^41^ Policies could be directed at ensuring nurturing environments for children, including early childhood programs, reducing adverse childhood experiences (poverty, violence, abuse, and neglect), and improving parenting skills.^42^

This study had important strengths and limitations to consider. It used a population-based cohort and included all records of physician-diagnosed mental disorders and of adverse adult outcomes. However, our study did not capture those who have experienced mental disorders during childhood or adolescence but were not seen by a physician. We also acknowledge that 23.2% of individuals were excluded because of lack of continuous health records due to leaving the province (Figure 1). This limits the finding's generalizability because individuals who left the province may be systematically different than those who lived in Manitoba throughout their childhood. A notable strength was our ability to adjust for other childhood factors that could potentially influence the adverse adult outcomes. Our analyses showed that these other childhood factors partially explained the association between childhood/adolescent mental disorders and adult outcomes but we certainly did not account for all confounders. Important characteristics such as smoking, social media use, and bullying were not captured. We note that society's understanding of childhood and adolescent mental disorders has improved rapidly over the last few decades and this may have influenced our results.^43^ For example, children and adolescents growing up in the 1990s may not have received adequate treatment for their mental illness. Future research could investigate further how early intervention and treatment influences long-term outcomes of children and adolescents experiencing mental disorders.

### Conclusion

This population-based longitudinal study showed that mental disorders diagnosed in childhood and adolescence appear to be important risk factors for a range of adult adversity. Risk of persistence underscores their chronicity, and their association with low income, social adversity, and justice system involvement emphasizes their impact on functioning. Given that many services touch the lives of children, efforts to promote mental health and prevent mental disorders require concerted efforts from multiple sectors including public health, child welfare, education, and justice systems. This enhanced knowledge could directly inform policy and practice to provide better population-level mental health promotion, prevention, and early intervention for children and adolescents with mental disorders to improve adult outcomes in the future.
